# The underexplored effects of economic transition on intellectual property rights protection: An economic geography perspective

**DOI:** 10.1007/s11192-025-05352-9

**Published:** 2025-07-16

**Authors:** Xing Gao, Senmao Xia, Yu Xiong, Xiaoxian Zhu, Yantao Ling, Mengqiu Cao

**Affiliations:** 1 Beijing Institute of Technology, Beijing, China; 2 University of Surrey, Guildford, UK; 3 Teesside University, Middlesbrough, UK; 4 Chongqing University of Technology, Chongqing, China; 5 University College London, London, UK; 6 Threshold of Social Sciences, Beijing Institute of Technology, Zhuhai, China; 7 National Innovation Institute for Small and Medium Sized Enterprise of China, Beijing Institute of Technology, Beijing, China

**Keywords:** Intellectual property rights protection, Knowledge management, Economic transition, Multi-level modelling, Huaihai economic zone, O34, O38

## Abstract

As an institutional guarantee of technology and innovation, intellectual property rights (IPRs) protection at the national and supranational level has long been an important focus of economics and politics. However, very few studies have examined IPRs protection in the fields of geography and urban studies. Thus, this study aims to investigate IPRs protection within evolutionary economic geography (EEG) by highlighting the effect of economic transition. Taking Huaihai Economic Zone (HEZ), in China, as a sample, the study uses spatial multi-level modelling to better understand the impacts of the threefold process of economic transition (i.e., decentralisation, marketisation and globalisation) on IPRs protection. Our analysis reveals important new insights including: (1) the horizontal spatial distribution of IPRs protection is uneven both horizontally and vertically, and it has significant spatial hotspots; (2) The driving force of China’s internal marketisation and decentralisation policy positively influences IPRs protection, unlike in the Global North, because strong IPRs protection is not suitable for the economic conditions of countries in the Global South due to the negative effects of globalisation; (3) Economic transition has a major influence on IPRs protection at the prefectural level, but not at the provincial level. The contributions of the study are twofold: theoretically, it is one of the first paper to examine IPRs protection at the sub-national level within the framework of EEG, and to use the triangular process of economic transformation to explain the resulting institutional changes. Methodologically, based on the theoretical underpinnings of our study, we take different administrative levels and autocorrelation into consideration in our model.

## Introduction

A substantial amount of literature has focused on investigating the effects of economic transition in transitional nations, including China, Eastern Europe, Russia, and Vietnam (Oi, 1992; Wu, 2016). Dramatic economic restructuring and transition have generated insights for exploring regional inequalities at different geographical scales. Relationships between different levels of government, the applicability of the market economy, and integration into globalisation are key points in the process of economic reform in every transitional country. It should also be borne in mind that some elements of economic transition and institutions are interdependent and interlinked. Exploring such complex interactions is of significance for the successful implementation of planning and global economic recovery, because it offers greater opportunities to different sectors to promote policy coherence and, thereby, the co-achievement of different targets (Laplume et al., 2014a, 2014b; Zhou & Tang, 2020). Although many studies have been conducted into the role of the economic transition pattern within the global, national and local economy (Sun, 2019; Wei et al., 2016), very few have examined the association between economic transition and institutions. Therefore, this study takes a new institution—intellectual property rights (IPRs) protection—and explores the effects of China’s economic transition on IPRs protection at regional and urban levels.

IPRs protection is regarded as an institutional driver of technological progress and economic growth. Existing studies have mainly focused on trying to explain the specific conditions that can change the intensity of IPRs protection. In particular, they have explored the roles played by international trade and investment, technological progress, innovation, institutions, and industrial agglomeration in regard to IPRs protection at a national level (Eicher & García-Peñalosa, 2008; Lin and Alon, 2019; Uchida, 2020). For example, Schneider (2005) concluded that IPRs protection is positively related to innovation in developed countries but has negative impacts in developing countries. However, Allred and Park (2007) questioned this conclusion, because they found there were no significant effects of IPRs protection on innovation in developing countries. In addition to the relationships between IPRs protection and innovation, some studies have explored the direct relationships between IPRs protection and national economic growth. For instance, based on data from developing countries, Chen and Puttitanun (2005) showed that there is a nonlinear relationship between IPRs protection and GDP level. Some studies have also tested the relationships between IPRs protection and economic growth in various countries and confirmed that there is a positive correlation (Gould & Gruben, 1996; Thompson & Rushing, 1996).

However, there has been little investigation into IPRs protection at regional and urban levels. This dimension is important as the intensity of IPRs protection varies considerably across different regions and administrative levels in China. IPRs protection has generally been implemented at a national level. However, in China, central government policies are often vague. As long as they do not violate central government policy, local governments are able to formulate policies based on the existing local conditions, and thus there are significant differences in the intensity of IPRs protection between various regions in China. Moreover, as economic decentralisation has progressed, the responsibility for economic growth has become increasingly detailed, resulting in greater competition and inequalities between different regions and cities (Wu, 2016). With regard to IPRs protection, different regions have different incentives and preferential policies for patent technology, as well as differing penalties for infringement. Overall, China’s IPRs protection and economic growth tend to be stronger in the east and weaker in the west. Despite this, there is a scarcity of studies that have investigated regional or urban IPRs protection in China. Qiu et al.(2016) assessed IPRs protection at the provincial level in China, and found that it had significantly negative impacts on the efficiency of investment in creative enterprises. However, they only used the rate of patents not being infringed to measure the intensity of IPRs protection. Although the patent rate is an output measurement of economic growth (Weinhold & Nair-Reichert, 2009), it is unable to reflect the way in which IPRs institutions enforce the law, and neither can it cover every kind of IPRs, such as trademarks, trade secrets, etc.

Linking to evolutionary economic geography (EEG), recent literature has tended to view locally related activities as promoting regional growth, and has classified these activities based on existing local capabilities and conditions, such as technological development (Heimeriks & Balland, 2016), spatial scales (Gao & Zhai, 2021), and time (Boschma, 2016). Thus, the regional diversification of institutions is a key focus of evolutionary economic geography (EEG). From an EEG perspective, a successful economic transition in a region requires the support of existing local activities and capabilities (Dawley et al., 2015). Consequently, institutions are necessary to support the process of economic transition (Dawley et al., 2015) and existing studies have verified the role of such institutions on a national scale (Boschma & Capone, 2015; Cortinovis et al., 2016), although they have not specifically focused on IPRs protection. Moreover, recent studies such as those by Wang et al. (2015) and Yi et al. (2015) have explored how region-specific institutions and the marketisation index affect economic transitions and IPRs protection, highlighting the complex interplay between globalisation and local institutional frameworks. The fact that the role of institutions at the micro-level or sub-national level has been ignored constitutes a significant shortcoming of such studies. According to the concept of the Window of Locational Opportunity (WLO) (Storper & Walker, 1989), when economic transition cannot be achieved via locally available capabilities, it becomes necessary for institutions to step in at the local level (Laplume et al., 2014a, 2014b). This is because economic transition creates specific economic and institutional conditions at a local level (Zhou & Tang, 2020). Thus, an important function of EEG is to shed light on institutional changes from a micro-perspective or at a sub-national level. In addition, although EEG emphasises the importance of innovation and technology, it has rarely been used to gain insight into the institutional mechanisms behind innovation and technology, namely IPRs protection. Therefore, this study adopts an EEG perspective to discuss how economic transition and IPRs protection vary in terms of spatial distribution at the sub-national level, rather than focusing solely on the national level.

However, previous studies have some limitations. First, to the best of our knowledge, no studies have used a systematic economic transition framework to analyse IPRs protection at the sub-national level. Although some studies have drawn various conclusions about the relationships between economic transition and IPRs protection in China, they have tended to treat the economic transition as part of China’s historical background (Feng, Kang and Chen, 2016; Yu, 2015), without clearly explaining what it involves and how it affects IPRs protection at the regional level. Second, there is a scarcity of studies on the role played by spatial analysis and urban differences with regard to IPRs protection. If we do not consider the spatial effects in the process of studying the institutional differences between cities or regions, we cannot observe the links between cities or regions, which is not conducive to economic integration and narrowing the gap between regions. Spatial analysis can help to overcome the administrative barriers as well as the obstacles posed by contradictory local interests, and thus promote effective cooperation and coordinated development between regions. Third, existing studies do not appear to make any distinctions between the effects of different administrative levels and nor do they explore the scale effects within regions. The administrative level not only refers to the implementation strength and spatial scope of institutions, but also the political, economic and social resources that the institutions can obtain. Thus, distinguishing between administrative levels can help us to detect more accurately which factors are reshaping IPRs protection at different spatial scales.

Therefore, this study aims to explore how economic transition affects IPRs protection at different spatial scales, using Huaihai Economic Zone (HEZ) as a sample case. Building on recent literature on economic transition (Huang et al., 2015; Wei, 2001), we employ a threefold process, consisting of decentralisation, marketisation and globalisation, as the proxy for economic transition. Conventionally, advanced economic development has been regarded as positively related to strong IPRs protection. However, our study challenges this view by considering the role of spatial heterogeneity in regional economic transition, and argues that successful economic transition does not always require strong IPRs protection. Overall, our findings suggest that the manifold mechanisms of IPRs protection are sensitive to differing spatio-temporal scales, and that IPRs protection plays an important role in regard to urban and technological innovation in the context of economic geography and urban planning.

This study makes two main contributions to the growing body of literature on the spatial and geographical analysis of economic and institutional issues. Theoretically, we extend the EEG framework to the sub-national level by examining IPRs protection at multi-level spatial scales, and thus explore another important dimension, in addition to politics and economics, that affects the intensity of IPRs protection: urban geography. In other words, we challenge the current assumption that advanced economic development is always positively related to strong IPRs protection by considering urban geography (Boschma & Capone, 2015; Cortinovis et al., 2016). From an EEG perspective, the study discusses the horizontal and vertical spatial diversification of economic transition and IPRs protection, and shows that the interactions between economic issues and political institutions have specific spatial characteristics on different scales (Laplume et al., 2014a, 2014b; Storper & Walker, 1989). On this basis, we also use the threefold process of economic transition to explain changes that have occurred in relation to IPRs protection. Through a multilevel analysis, we provide a more comprehensive understanding of how IPRs protection varies not only across different regions and cities but also across various administrative levels (Zhou & Tang, 2020). Specifically, horizontal spatial diversification refers to the differences between regions and between cities, while vertical spatial diversification as a result of decentralisation pertains to the differences between administrative levels, i.e. national, provincial, and municipal levels. By incorporating these dimensions, we can enhance our understanding of how local contexts and administrative structures shape the effectiveness of IPRs protection. This approach not only broadens the application of EEG but also provides a more nuanced view of the spatial dynamics at play. Thus, our discussion of this topic within an EEG framework also guides our strategic choice of empirical analysis.

Empirically, based on the specified theoretical considerations, the study first maps the spatial patterns of IPRs protection on different spatial scales, in order to show the institutional spatial differences between cities and between regions regarding the IPRs protection regime. Furthermore, the decentralisation process that accompanies economic transition makes multi-level analysis the most appropriate model for examining vertical diversification, while horizontal diversification suggests that there are autocorrelation issues that need to be dealt with. Spatial analysis can be used to overcome the administrative barriers associated with conflicts and disparities between local interests and achieve more effective cooperation and coordinated development between regions. It is helpful for enhancing horizontal collaboration between different municipal governments, thereby promoting policy uniformity and regional integration (Cortinovis et al., 2016; Zhou & Tang, 2020). In addition, distinguishing between different administrative levels can help to identify which factors influence IPRs protection on different spatial scales. For the sake of robustness, we address the issue of endogeneity in relation to economic transition and IPRs institutions using multiple spatial scales. Overall, our theoretical considerations and empirical analysis strategy are closely connected and reinforce each other.

## Theoretical considerations

### Exploring economic transition and IPRs protection within the framework of EEG

The EEG literature claims that the effects of economic transition on IPRs protection are spatially defined. In other words, diversification is emphasised from a regional perspective (Boschma et al., 2017). Consequently, the spatial differences in regard to economic transition and IPRs protection between regions and cities are the focus of this study. Economic transition creates challenges for existing institutions (Stinchfield et al., 2013). If economic transition is unrelated to the IPRs protection capability that already exists within a region, then it is not suitable for the local conditions in that area. As the economic transition process evolves it would lead to unrelated diversification, which includes place dependence and path dependence in the mechanisms of economic transition that affect IPRs protection (Heimeriks & Boschma, 2014). Economic transition is influenced by a dependence on the cycle of local economic reproduction with respect to localised knowledge, institutions and vested interests. This means that the economy obtains the resources that it uses from the surrounding natural environment and produces various material products through labour, while some of the unused waste resources generated are discharged back into the natural environment to perpetuate the cyclical process (Ron Boschma et al., 2017). The IPRs protection regime tends to be nationally led, and this is reflected in different regions of a country. However, the IPRs protection regime is neither fully a globalised monolithic nor a monolithic one (Fuenfschilling & Binz, 2016). That is, it may be weak in some regions, but stronger in others, because it is affected by local conditions in regard to the diversification associated with economic transition. Consequently, the extent to which the strength of IPRs protection is regionally institutionalised differs between regions (Crouch & Voelzkow, 2009). Thus, the combination of local conditions and economic transition can reshape IPRs protection, under the framework of EEG.

Furthermore, the diversification inherent in the evolutionary processes is not only reflected in the differences between regions at the horizontal level, but also in the differences between various administrative levels in a vertical sense. A new IPRs protection regime implemented in response to economic transition may be new to a city, but it is not necessarily new to the province in which the city is located. Because there are differences between the local conditions found in provinces and cities, the evolutionary processes of economic transition and IPRs protection also differ at the vertical geospatial level (Beer et al., 2019). Therefore, it can be best understood from an EEG perspective, which also fits with the empirical analysis strategy of the spatial multi-level model used in our study. According to EEG, weak IPRs protection can rapidly unravel any economic interests linked to a geographical network, especially in emerging and undeveloped regions (Rodríguez-Pose & Di Cataldo, 2015; Rodríguez-Pose & Zhang, 2020). Thus, the evolutionary processes of economic transition that affect IPRs protection at different spatial scales need to be examined, to prevent economic transition being derailed by an unfavourable IPRs protection regime (Rodríguez-Pose, 2021). Overall, the EEG perspective facilitates a better understanding of spatial distribution and evolutionary differences in economic activities and institutional change at both the horizontal and vertical dimensions of diversification. In addition, gaining a greater understanding of the relationships between economic transition and IPRs protection can help to shed light on how local economies and corresponding institutions evolve, become transformed, and enter new stages in response to local existing conditions.

### How economic transition affects IPRs protection

**Fig. 1:**
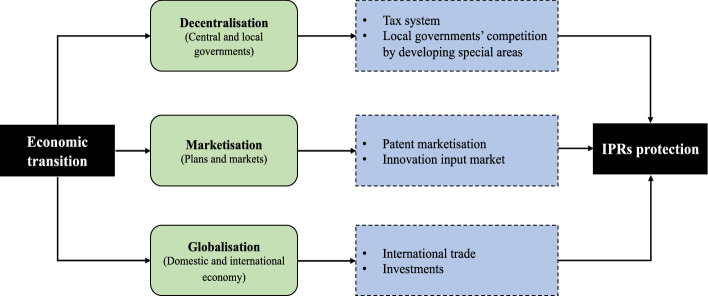
Framework for economic transition and IPRs protection

China’s economic transition is characterised by the following three relationships: between central and local governments (decentralisation); between a planned and a market economy (marketisation); and between the domestic and international economy (globalisation) (Huang et al., 2015; Wei, 2001). Previous studies on China's economic transition have only focused on the transition from a planned to a market economy (Shin, 2016; Yeh et al., 2015), but they have ignored the impact of the international economy and decentralisation on economic transition. Thus, the threefold framework described above can be helpful in terms of understanding more about the evolutionary processes associated with China's economic transition, and we use it in this study to gain insight into changes in IPRs protection. Figure 1 shows the framework that can be used to illustrate the relationship between China’s economic transition and IPRs protection. By investigating China’s experience of economic transition and radical economic spatial restructuring over recent decades, focusing specifically on the sample case of HEZ and emphasising the presence of multi-level spatial scales, we can gain a better understanding of the debates about whether the intensity of IPRs protection should be strengthened in the Global South. In addition, we explore the unique institutional change mechanism involved in the process of China's economic transition, and illustrate the coexistence of economic success and undeveloped institutions. These processes show how the formation of socialist national institutions is related to the transformation of the urban spatial economy.

Decentralisation from the central government to local governments has dramatically changed the behaviour of local governments and relationships between central and local government in China (He & Zhu, 2007). Consequently, local governments now have much greater responsibility for regional economic growth in their respective jurisdictions (Qian & Weingast, 1996). The main aspects of decentralisation that affect IPRs protection are the tax system and competition between local governments. China’s tax sharing reform of 1994 changed the fiscal allocation relationship between the central and local governments. To try to redress the growing financial gap between different regions, the central government further reformed the income tax sharing system in 2002. These reforms effectively changed local governments’ sources of revenue, and increased the proportion of tax received by central government at the expense of local governments (Huang et al., 2015). In order to promote regional economic growth and revenue, local governments therefore have to seek extra-budgetary revenue (Eckaus, 2003). Thus, attracting investment has become an important tool for boosting regional economic growth. Because compensation fees for farmers are low, local governments can acquire land from them on which to build economic and technological development zones or high-tech industrial parks. These pieces of land are then sold to enterprises on a large scale, which can significantly increase revenue for local governments. However, to retain these enterprises and ensure a continuous flow of revenue, local governments have to provide appropriate IPRs protection (Abbas et al., 2018). They can do this, for example, via preferential IPRs policies, levying new taxes on IPRs, and offering special patent protection to these enterprises (Yao & Zhu, 2006). Moreover, depending on local conditions, local governments have the right to set up their own IPRs protection projects. These kinds of local IPRs protection not only attract more investment, but also increase local fiscal revenue.

IPRs protection is also affected by competition between local governments with regard to decentralisation. By establishing special areas within a city such as economic development zones or industrial parks, this mainly takes the form of competition for investment (Huang et al., 2015). As the sole supplier of local IPRs protection, local governments can use their IPRs protection as a vital instrument for attracting investment (Feng & Kang, 2015). In China, local governments at all levels formulate IPRs protection strategies in line with their actual interests in their special areas. In addition, to attract more investment, local governments tend to increase investment in research and development (R&D) and fiscal expenditure in these special areas, which can promote innovation and improve IPRs protection (Jandhyala, 2015). As local governments are not allowed to adjust tax rates to offer tax breaks and waivers (Ding, 2007), they focus their efforts on reaping the benefits of increasing fiscal expenditure on science and technology, thereby promoting innovation instead. The former can have the effect of increasing IPRs assets (Sebrek, 2020). Thus, local fiscal expenditure may have a positive effect on IPRs protection (Gao & Zhai, 2018).

Marketisation is an important part of China’s economic transition, and it has been strengthened since China joined the WTO. Due to economic growth and the expansion of trade, marketisation has reduced the intensity of IPRs protection (Boldrin & Levine, 2009). By contrast, under the influence of market mechanisms, patent protection has strengthened the direction, rather than the scale, of R&D investment (Kumar, 1996). Furthermore, the market power across regions may promote IPRs protection in countries in the Global South (Saggi, 2013). Specifically, marketisation affects IPRs protection through patenting and innovation by private enterprises. China’s economic transition is driven by market-oriented economies, and the market is regarded as a tool for distributing goods and production factors (He & Zhu, 2007). In terms of economic and spatial development, weak state regulation and strong market mechanisms can affect aspects of patenting and innovation among private enterprises. As IPRs are a product of the market economy, the use and mobility of IPRs protection is guided by market mechanisms. The market value of patents also determines the extent to which IPRs need to be protected. Moreover, in the era of the knowledge economy, the transfer of patents has become less straightforward and more diversified, driven by market mechanisms, which has opened greater possibilities for realising the value of IPRs protection (Guo, 2017). In addition, to maintain competitive advantage, firms tend to devote more time and effort to patenting and patent protection than they did previously (Lu & Liu, 2008). As a feature of marketisation, enterprise reform has introduced market mechanisms into the realm of IPRs protection. Following the aforementioned reforms, most companies have transformed themselves from socialist units (danwei) into profit-seeking and market-oriented firms (Wei, 2000) and, driven by marketisation, they have had to develop more innovative strategies for making profits.

Globalisation influences IPRs protection through international trade and investments. Against a background of economic globalisation, the relationship between international trade and IPRs protection has become increasingly significant (Campi & Dueñas, 2019). Participating in global trade has compelled China to establish its current IPRs system. In order to attract more trade and open up international markets, local governments have been very keen to establish a ‘Pilot Free Trade Zone’ in an effort to promote local development and improve the efficiency of IPRs protection (Shang, 2017). Globalisation has influenced every aspect of contemporary China’s urban institutions via investment (Wei, 2012). First, foreign direct investment (FDI) can alleviate the financing constraints on local enterprises, and promote regional innovation and IPRs protection (Javorcik, 2004; Liu, 2016). Due to the aforementioned difficulty in obtaining credit domestically, some private enterprises have become willing to cooperate with multinational corporations (Huang, 2001). Thus, these enterprises seek stronger IPRs protection. Second, FDI impacts on IPRs protection via technology spillover. Advanced technology is transferred to the host country along with FDI, and thus FDI speeds up the general transfer speed of technology within a region, and promotes technological progress and knowledge accumulation in the area (Sun, 2008). Technology spillover may also encourage local enterprises to learn more about advanced management techniques (Liu, 2016), which in turn promotes greater awareness and increases the intensity of IPRs protection within a region.

## Methodology

### Data

**Fig. 2:**
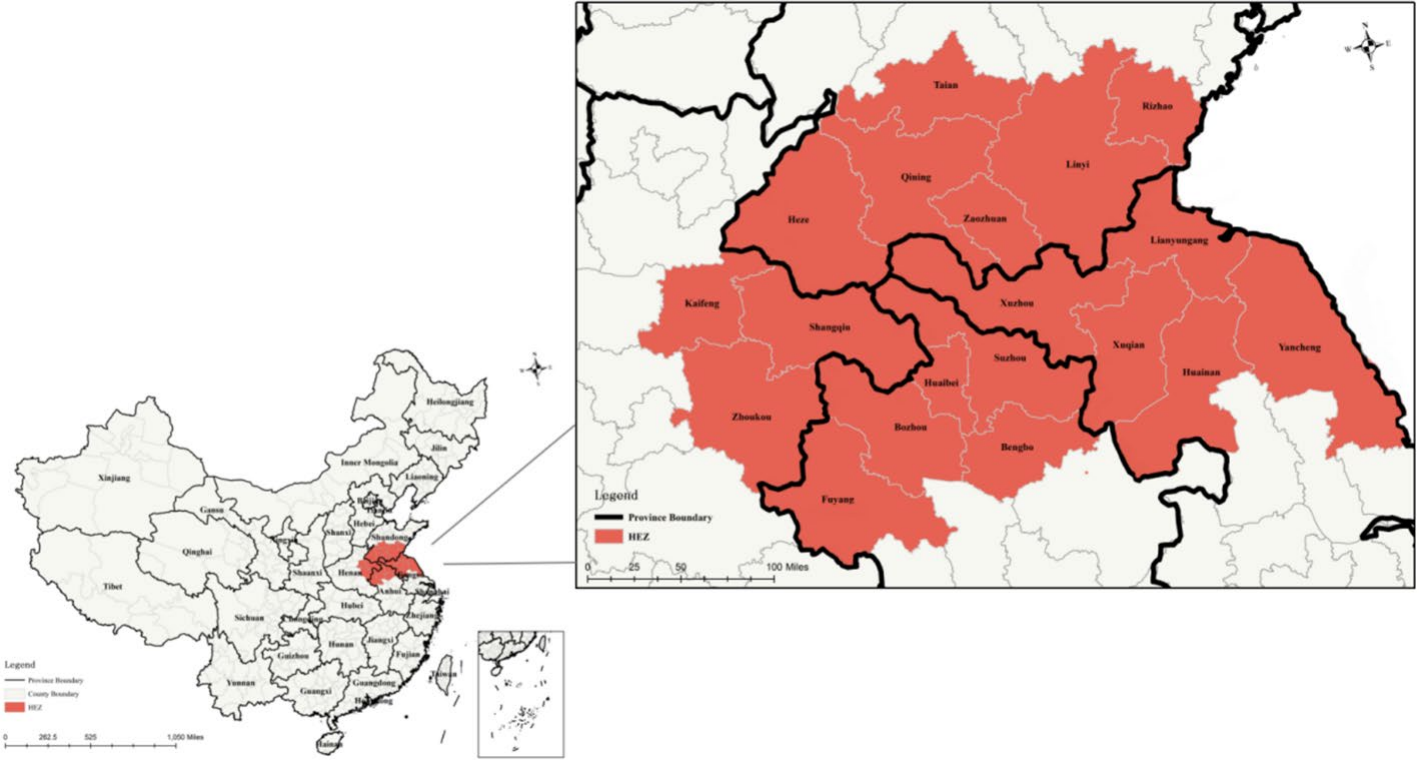
The location of HEZ

HEZ consists of 20 prefecture level cities which border four provinces, namely: Jiangsu, Shandong, Henan and Anhui. However, Laiwu has recently been downgraded and merged into Jinan, so we excluded it from our analysis. A total of 19 prefecture-level cities were included in the study (Fig. 2).The land area occupied by HEZ accounts for 1.86% of the whole country – an area larger than England. Its resident population was 56 million in 2021, which is larger than that of Australia. The cities in HEZ are very similar in terms of natural resources, history of development, and cultural habits, and thus it is a relatively stable and complete regional unit (Zhou et al., 2010). We chose HEZ as a sample case for the following reasons:

First, because of the immature status of IPRs protection in HEZ; IPRs is not institutionalised to the same degree across regions or cities. The conditions in HEZ therefore provide an opportunity to explore the spatio-temporal pattens of the IPRs institution, which can provide a useful point of reference for the promotion of regional economic transition. Second, the case of HEZ can helps to explain how an economically developing region creates suitable IPRs institutions and how IPRs institutions can be utilised to promote economic transition. Based on local conditions, appropriate IPRs protection can be used to promote the spatial agglomeration of aspects of innovation, thereby accelerating economic transition within the area. Third, the topic is in line with HEZ’s official planning strategy. This planning strategy could contribute to the implementation of unified IPRs protection across cities, which has the potential to positively affect regional integration and economic transition as well as improving governance (McFarlane et al., 2016). Using our sample, we aim to provide insights into the protection of IPRs across China. China has made significant strides in strengthening its IPRs regime, especially with the implementation of various laws and regulatory reforms. For instance, the 2017 amendments to the Patent Law and the establishment of specialised IPRs courts have been crucial steps in this direction (Zhuang et al., 2020). However, there remain considerable disparities in IPRs protection at the provincial and city levels, which this study aims to highlight. By examining the case of HEZ, we can better understand these differences and identify the gaps that need to be addressed to foster a more robust IPRs environment nationwide (Dai & Sun, 2025).

Our sample period runs from 2011 to 2020. The data on the provinces were obtained from China’s and its provinces’ Statistical Yearbooks, while the data on prefectural cities came from the Statistical Yearbooks of 19 prefectural cities in the HEZ. Choosing 2010 as the base year and using that year's consumer Price Index (CPI) to deflate the data for 2011–2020 helped to ensure comparability of the data over time series. This method excludes the effect of inflation and allows direct comparison of currency values from year to year (Gibson et al., 2008).

### Variables

#### Dependent variable

The dependent variable used in this paper is IPRs protection measured at city level based on the method used by Han and Li (2005). Originally based on Ginarte and Park’s (1997) (G-P) IPRs index, we used the quantitative index developed by Han and Li (2005). It is important to note that law enforcement has been used to refine and improve the G-P method. First, the method grasps the fact that the law is the foundation of IPRs protection. The G-P method has been referenced over 1,003 times (Cassandra & Dalibor, 2015), and is completely based on the legal institutional system. Thus, it is able to capture a broader range of characteristics relating to the variability in IPRs protection across administrative boundaries (Maskus & Penubarti, 1995). The fact that the method incorporates law enforcement enhances the G-P method because the latter only evaluates whether the IPRs protection law has been formulated, but does not consider the specific effect of its implementation (Weinhold & Nair-Reichert, 2009). Moreover, it is only suitable for applying to cases of developed countries with relatively sound judicial systems. Thus, adding law enforcement to the G-P method enables the intensity of IPRs protection to be assessed.

However, some other studies have chosen various different methods to measure IPRs protection at sub-national level, but none of them meet the two requirements specified above at the same time. For example, Ang et al. (2014) constructed two novel measures of regional IPRs protection, one based on plaintiff win rates in provincial courts and the other based on the frequency with which IPRs are mentioned in Chinese official newspapers. These two measures seem to be moving increasingly far away from the law itself, and it is also difficult to apply them on a smaller spatial scale. In addition, survey data have also been used to quantify IPRs protection at prefecture level (Fang et al., 2017). However, surveys are based on individual subjective perceptions, and the range and population groupings of respondents is limited. Therefore, it is also difficult for this method to reflect the objective intensity of IPRs protection. In addition, it is difficult to distinguish whether a respondent represents a municipal level or provincial level, so it is unsuitable for a multi-level study. Lastly, some academic organisations or institutions also include IPRs protection in their own market survey reports, such as the NERI index developed by Fan et al. (2011). In fact, the measure used in the report neither involves the law itself and nor does it reflect how the institution is implemented. Moreover, there is a strong likelihood of endogeneity issues occurring in the economic modelling analysis. Therefore, the measure that we used has the ability to fully reflect the objective strength of IPRs protection and can be applied on different spatial scales, which is consistent with our aim. The equation for IPRs protection is shown below:

1 $${P}^{A}\left(t\right)=F\left(t\right)*{P}^{G}\left(t\right)$$

where,$t$ is time, and ${P}^{A}\left(t\right)$ is the strength of IPRs protection; ${P}^{G}\left(t\right)$ is the strength of IPRs protection measured by the G-P method; $F\left(t\right)$ is law enforcement efforts. The value of law enforcement efforts ranges from 0 to 1. 0 signifies that the provisions of IPRs protection, regulated by law, have not been implemented at all, whereas 1 denotes that they have been fully implemented.

Four indices were selected to measure law enforcement efforts (Han and Li, 2015; Gao, 2020): 1) The legalisation of society, measured by the lawyer ratio: where the ratio of lawyers to that of the total population is over five in ten thousand, the value of the legalisation of society is 1, or otherwise the value is derived by dividing the lawyer ratio by five ten thousandths; 2) Refinement of the legal system, measured by legislative time: if legislative time is over 100 years, the value is 1, or otherwise the value is obtained by dividing real legislative time by 100; 3) Economic development, measured by per capita GDP: when per capita GDP is over 1,000 dollars, the value is 1, or otherwise the value is obtained by dividing real per capita GDP by 1,000 dollars; 4) The supervision and balance of international society, measured by whether the country is a member of the WTO: if it is, the value is 1, and if it is not, the value is 0. We measured the aforementioned four variables at the provincial and prefectural level for HEZ. Because China is a centralised country and became a member of the WTO in 2001, the values for the supervision and balance of international society from 2011 to 2020 are always 1. Therefore, during the period from 2011 to 2020, this value remained constant and will not have any effects. Thus, we removed it from the measure of IPRs protection. The final scores for *F(t)* are the arithmetic means of the four indicators. Overall, this measure focuses on legal protection.

#### Independent variables

A series of independent variables was used to conceptualise China’s IPRs protection as a threefold process of economic transition, and the multi-level analysis was used to reflect decentralisation. As globalisation has accelerated, the system of technological protection and economic growth has been boosted by increasing FDI, particularly in open coastal cities (Huang et al., 2015). FDI promotes the use of technology and patent invention via the technological spillover effect. In order to maintain the value of patents and technology, transnational corporations encourage stronger IPRs protection in host countries (Arghya et al., 2018). This study uses the ratio of FDI to GDP (FDI) to represent the impact of globalisation. In addition, international trade was also used to reflect the extent of globalisation. The Global North advocates a stronger IPRs protection regime than China would be able to afford (Babovic & Wasan, 2011). Moreover, international trade can affect economic growth through the IPRs regime (Wu et al., 2013). Therefore, the proportion of total export–import volume to GDP (Trade) was selected to measure the influence of international trade. The ‘Coastal_City’ variable was selected to indicate whether a city is coastal. Coastal cities receive more policy support in order to attract FDI, and have a natural advantage in terms of international trade.

Regarding marketisation, the roles of private enterprises and markets are particularly emphasised. China's economic reform has spawned a large number of private enterprises. Meanwhile, a series of economic policies have been introduced to encourage private enterprises to innovate, which has increased market vitality. Thus, the ratio of innovation expenditure by private enterprises to fiscal revenue (IEPF) was chosen. Economic marketisation also involves patent marketisation, as only authorised patents have the opportunity to achieve their market value and bring real benefits. Therefore, they can be used as an effective measure of the output of market innovation (Weinhold & Nair-Reichert, 2009). Additionally, patent life cycle theory suggests that patents and technologies may need stronger IPRs protection in order to reap more benefits (Zhang et al., 2024). Thus, the ratio of patents granted to private enterprises to the total number of patents granted (PPGT) in each prefectural city was selected.

Decentralisation is the key reason that this study chose to employ multi-level analysis. Decentralisation is the process by which authority is shifted from upper-tier governments to lower-tier governments, which are more attuned to local circumstances and preferences (Boisot & Meyer, 2008). Local governments treat cities as facilitators of innovative activities. Without local governments playing an active role as innovators, public sector innovations may not have occurred. Thus, local governments can contribute to regional innovation by promoting stronger IPRs protection (Teemu et al., 2018). In addition, local governments have focused on the exceptional value of IPRs and viewed it as a sustainable source of local finance. Therefore, local governments usually try to increase fiscal revenue by promoting IPRs protection. Unlike other studies, ours refines the measure of IPRs protection at regional level to include prefectural decentralisation and provincial decentralisation. Doing so helps to offer a better understanding of the impact of decentralisation on IPRs protection in transitional China. Provincial decentralisation involves the transfer of power from China’s central government to its provincial governments, while prefectural decentralisation entails the transfer of authority from provincial governments to prefectural governments (Huang et al., 2015).

Following Huang et al. (2015), we use the proportion of fiscal expenditure at low levels compared to that at higher levels to measure fiscal decentralisation. Thus, we chose two groups of variables with which to measure decentralisation: (1) Decentralisation level of fiscal expenditure (DLFE). (2) Decentralisation level of fiscal revenue (DLFR). This measure can be represented as follows:

2 $$DLFE_{pre} = \frac{{FE_{pre} - FTP }}{{FE_{pre} - TP}},\,DLFE_{pro} = \frac{{FE_{pro} - FTP }}{{FE_{pro} - TP}}$$

3 $$DLFR_{pre} = \frac{{FR_{pre} - FTP}}{{FR_{pre} - TP}},\,DLFR_{pro} = \frac{{FR_{pro} - FTP}}{{FR_{pro} - TP}}$$

where, ${FE}_{pre}$ is total fiscal expenditure at prefecture level; ${FE}_{pro}$ is total fiscal expenditure at provincial level. ${FR}_{pre}$ is total fiscal revenue at prefecture level; ${FR}_{pro}$ is total fiscal revenue at provincial level. $FTP$ is financial transfer payment from provincial government to prefecture level; and $TP$ is transfer payment between governments.

Some research considers decentralisation in terms of the efficiency of legal enforcement (Li et al., 2015; Wang et al., 2015). Administrative decentralisation has resulted in provincial governments wielding considerable judicial autonomy, often impacting on court rulings (Peck & Zhang, 2013). Although the measurement that evaluates the efficiency of legal enforcement has its advantages, particularly in capturing the effectiveness of judicial systems in upholding the rule of law, it is not entirely suitable for the context of our research. The primary reason is that our study focuses on the multifaceted nature of decentralisation and its impact on regional innovation and IPRs protection in China. The efficiency of legal enforcement, while important, is but one aspect of the broader institutional framework that influences IPRs protection. By focusing solely on the efficiency of legal enforcement, we would overlook the complex interplay between political, economic, and administrative factors that shape the protection of IPRs at the regional level.

(3) Economic and Technological Development Zones (ETDZ). ETDZs are instrumental in enhancing the economic conditions of the cities where they are established (Jia et al., 2020). They can invigorate regional economic dynamism by attracting investment and expanding business opportunities (Wang et al., 2022). In China, ETDZs or high-tech industrial parks operate at varying administrative tiers, with those at higher levels receiving greater financial backing (Zhuang & Ye, 2023). National economic and technological development zones are pivotal in China's pursuit of innovation-driven development, nurturing high-tech industries (Schminke & Biesebroeck, 2013). Consequently, we employ the presence of national economic and technological development zones or high-tech industrial parks as an indicator for evaluation; (4) ‘Central’ is a dummy variable, which tests whether a city is a provincial capital, a sub-provincial city or a central city within the region.

The second group of variables relates to the provincial level and comprises the following four variables. (1) DLFE _Cent: this refers to the ratio of DLFE at provincial level compared to that at national level; (2) DLFR _Cent: this denotes the ratio of DLFR at provincial level compared to that at national level; (3) ETDZ_Cent: this is a measure of how many national economic and technological development zones and high-tech industrial parks there are within a province; (4) SP_Cent: this indicates whether a province contains one or more sub-provincial cities.

#### Control variables

Based on previous literature and endogenous growth theory (Henderson, 2004; Weinhold & Nair-Reichert, 2009), we also selected four control variables that could have an effect on IPRs protection, namely: urban population (Urbpopu), education (School), average wage per capita (WL), and the value of IPRs protection in the base year (Base). Table 1 summarises all the variables.

**Table 1:** Descriptions of the selected variables

Dimension	Variables	Definitions
Dependent variable	IPRs	The change in IPRs protection calculated by the equation: ${\text{P}}^{\text{A}}\left(\text{t}\right)=\text{F}\left(\text{t}\right)*{\text{P}}^{\text{G}}\left(\text{t}\right)$
Globalisation	FDI	The ratio of FDI to GDP at prefectural level
	Trade	The ratio of total export–import volume to GDP at prefectural level
	Coastal_City	Whether a city is coastal
Marketisation	IEPF	The ratio of innovation expenditure by private enterprises to fiscal revenue at prefectural level
	PPGT	The ratio of patents granted to private enterprises to total patent grants granted at prefectural level
Decentralisation at prefectural level Decentralisation at provincial level	DLFE	${DLFE}_{pre}=\frac{{FE}_{pre}-FTP }{{FE}_{pre}-TP}$
	DLFR	${DLFR}_{pre}=\frac{{FR}_{pre}-FTP}{{FR}_{pre}-TP}$
	ETDZ	Whether a city has national economic and technological development zones (ETDZ) or a high-tech industrial park
	Central	Whether a city is a provincial capital, a sub-provincial city or a central city within a region
	DLFE_Cent	${DLFE}_{pro}=\frac{{FE}_{pro}-FTP }{{FE}_{pro}-TP}$
	DLFR_Cent	${DLFR}_{pro}=\frac{{FR}_{pro}-FTP}{{FR}_{pro}-TP}$
	ETDZ_Cent	How many national economic and technological development zones and high-tech industrial parks there are in a province
	SP_Cent	Whether a province contains one or more sub-provincial cities
Control variables	Urban_Population	Proportion of population in urban areas at prefectural city level
	School	Average number of years’ schooling of adult (18 + years) population at prefectural city level
	WL	The average wage per capita at prefectural city level
	Base	The value of IPRs protection in 2010 at prefectural city level

### Methods

We used a multi-level model to examine the effects of economic transition on IPRs protection. First, an ordinary least squares (OLS) model at prefectural level was used to test whether the threefold process of economic transition can reasonably explain the change in IPRs protection in HEZ. Second, a single-level regression technique processes units of analysis as independent observations, which runs the risk of ignoring hierarchical structures (Li & Wei, 2010). Thus, the standard errors of the coefficients will be underestimated, and the statistical significance overstated (Subramanian et al., 2001). Because a multi-level regression model can integrate individual variables with unobserved heterogeneity at the aggregate level into a single model (Luis and Jose-Julian, 2018), we employed a multi-level regression model to assess the impact of both prefectural and provincial administrative levels. Third, because the topic is being explored from an EEG perspective, we used the spatial application of multi-level regression modelling to distinguish the impacts of prefectural characteristics from those of provincial characteristics. Data on changes in IPRs protection (e.g., 2016 data minus 2015 data) were used as the dependent variables. First, the basic OLS model is as follows:

4 $${IPRSP}_{tj}={\beta }_{0}+\sum_{k}{\beta }_{k}{EconomicTransition}_{ktj}+\sum_{n}{\beta }_{n}{C}_{ntj}+{\varepsilon }_{tj}$$

where, $t$ and $j$ represent time and prefectural city, respectively; IPRSP refers to the total change in IPRs protection; $EconomicTransition$ and $C$ denote sets of $k$ independent variables and $n$ control variables, respectively; ${\varepsilon }_{tj}$ denotes the error term of city *j* in *t* time.

Secondly, multi-level regression was carried out. The first level regression model used prefectural-level data, while provincial level data was added in the second model. Thus, the OLS equation then became:

5 $${IPRSP}_{jti}={\beta }_{0}+{\beta }_{1}{Decent}_{jti}+{\beta }_{2}{Market}_{jti}+{\beta }_{3}{Global}_{jti}+{\beta }_{4}{Decent\_Provin}_{jt}+{\beta }_{5}Year+ \sum_{n}{\beta }_{n}{C}_{njti}+{\mu }_{0t}+{\varepsilon }_{jt}$$

where, $i$ is the year, and $j$ and $t$ refer to the prefectural city and province, respectively; ${Decent\_Provin}_{jt}$ are the variables for each city $j$ in year $i$ at the provincial level; $Year$ refers to the dummy time variables and controls for time effects; $C$ represents a set of control variables; ${\mu }_{0t}$ and ${\varepsilon }_{jt}$ represent the error terms at provincial and prefectural levels, respectively.

However, third, due to the spatial spillover effects associated with economic development (Eapen, 2012), economic transition may be more advanced and IPRs protection stronger in a particular region than in surrounding regions. In addition, the value of economic transition for given links between variables may depend on the values for other links between variables by which they are connected, thus showing the autocorrelation between them (Kerkman et al., 2017). Therefore, spatial dependencies should be considered in the estimation to avoid biased results. In order to deal with the issues resulting from autocorrelation, the study employed a combined spatial error and lag model based on the method used by Kerkman et al. (2017). In this model, information about spatial dependence is included in a spatially lagged dependent variable, and the spatial lag model can be expressed as follows:

6 $$\left(IM-{\theta }^{2}{W}^{2}\right)IPRSP={{\beta }^{2}ET}^{2}+{\epsilon }^{2}$$

7 $$IPRSP={\theta }^{2}{W}^{2}IPRSP+{{\beta }^{2}ET}^{2}+{\epsilon }^{2}$$

where, $IPRSP$ is the vector of the change in IPRs protection between city *i* and city *j* with *(N* = n*n–n) matrix dimensions for given cities, while *-n* means that diagonal/intra-regional flows are excluded (*n* is 4 and 20 at provincial and prefecture level, respectively); IPRSP refers to a N*k design matrix for *k* explanatory variables with respect to economic transition; *W*^*2*^ is the network weight matrix of N*N; *β*^*2*^ represents the vector of parameters; ${\theta }^{2}$ is the parameters of network autocorrelation, and ${\epsilon }^{2}$ is the vector of errors at *N* dimensions. $IM$ denotes the identity matrix. Any spatial autocorrelation resulting from omitted explanatory variables (economic transition) can be treated by the error term (Kerkman et al., 2017). Thus,

8 $${\epsilon }^{2}=\sigma {W}^{2}{\epsilon }^{2}+\mu$$

where, $\sigma$ represents the estimated spatial error parameter; and $\mu$ is the random error term which is independent and identically distributed. In a spatial regressive model, the specification of the network weight matrix is important, because it can indicate a dependence structure between economic flows. The weight matrix used in the study is an inverse distance squared matrix. Given that the flow of established types of knowledge is limited by cost and geographical distance (Acs, et al., 2002), spatial physical features are included in the examination. Doing so can effectively reveal the spatial spillover from an economic geography perspective. The matrix was set according to the reciprocal of the distance between two cities as follows:

9 $${W}_{ij}=\frac{1}{{d}_{ij}^{2}}$$

10 $${\sum }_{i}{w}_{ij} =1$$

where, $i$ and $j$ refer to different provinces and cities. $d$ indicates the distance. Regarding all $i$ and $j$ (as long as $i\ne j$), ${W}_{ij}$ denotes the i-j_th_ element of $W$, and is the assumed strength of interaction from $i$ to $j$. In terms of the diagonal elements of $W$, we assume ${W}_{i,i}=0$ in order to avoid using another city’s level of IPRs protection to predict itself. Furthermore, spatial autocorrelation tests were applied to test for the presence of spatial autocorrelation among the observed values (Wang, et al., 2020). Therefore, we used Moran’s I to test the global spatial autocorrelation. Roberts and Goh (2011) suggested that using the same matrix to examine autocorrelation is most effective. The formulae for both are as follows:

11 $${\text{Global }}Moran^{\prime}s I = \frac{{n\mathop \sum \nolimits_{i = 1}^{n} \mathop \sum \nolimits_{j = 1}^{n} \omega_{ij} \left( {x_{i} - \overline{x}} \right)\left( {x_{j} - \overline{x}} \right)}}{{\mathop \sum \nolimits_{i = 1}^{n} \mathop \sum \nolimits_{j = 1}^{n} \omega_{ij} \mathop \sum \nolimits_{i = 1}^{n} \left( {x_{i} - \overline{x}} \right)^{2} }}$$

In this euqation, ${x}_{i}$ and ${x}_{j}$ represent the total number of IPRs for cities $i$ and $j$, respectively; $\overline{x }$ is the average total number of IPRs across cities; ${W}_{ij}$ is the spatial weight matrix, indicating the level of interdependence and association between spatial units. A positive or negative value represents a positive or negative spatial autocorrelation, respectively. The Global Moran's I provides an overall description of spatial autocorrelation for the entire study area, but is unable to shed light on the specific patterns and locations of these autocorrelations. The purpose of the Getis-Ord ${G}_{i}^{*}$ is to test whether a clustering tendency exists within the study areas (Wei et al., 2013). Therefore, we employed this method to help us identify the specific locations and intensities of these autocorrelations in space (Getis, 1992). The formulae for both are as follows:

12 $${G}_{i}^{*}(d)=\frac{\sum_{j}^{n}{W}_{ij}{X}_{j}}{\sum_{j}^{n}{X}_{j}}$$

13 $${Z(G}_{i}^{*})=\frac{{G}_{i}^{*}-{E(G}_{i}^{*})}{\sqrt{Var{G}_{i}^{*}}}$$

We used the hotspot analysis tool based on the Getis-Ord statistical index in the ArcGIS platform to carry out these calculation. In formula (12) and (13), ${E(G}_{i}^{*})$ represents the mathematical expectation; $Var{G}_{i}^{*}$ represents the coefficient of variation; ${W}_{ij}$ is the spatial weight matrix. When ${Z(G}_{i}^{*})$< 0, this indicates that the values surrounding the study area are below the mean, representing a clustering of low values, which are cold spots within the study area. When ${Z(G}_{i}^{*})$> 0, this tells us that the values surrounding the study area exceed the mean, representing a clustering of high values, which are hot spots within the study area.

Table 2 displays a summary of the descriptive statistics for the variables, while Table 3 shows the results of the Pearson correlation coefficient. A maximum value of no more than 0.7 proves the validity of the data.

**Table 2:** Summary of descriptive statistics

Variables	Minimum	Maximum	Median	Standard Deviation
FDI	0.002	0.367	0.070	0.118
Trade	0.010	0.982	0.132	0.211
IEPF	0.207	0.719	0.513	0.145
PPGT	0.116	2.317	0.344	0.447
DLFE	0.012	0.061	0.022	0.035
DLFR	0.011	0.211	0.013	0.025
DLFE _Cent	0.003	0.017	0.006	0.017
DLFR _Cent	0.071	0.869	0.531	0.518
ETDZ_Cent	1	27	12	4.281
Urbpopu	0.144	0.607	0.404	0.311
School	0.002	11.231	4.262	3.986
WL	1.249	4.852	2.073	2.147

A total of 2940 observations were used in the multi-level analysis

**Table 3:** Results of the pearson correlation coefficient

	IPRSP	FDI	Trade	IEPF	PPGT	DLFE	DLFR	DLFE _Cent	DLFR _Cent	ETDZ_Cent	Urbpopu	School	WL
IPRSP	1.000												
FDI	0.419^***^	1.000											
Trade	0.131^***^	0.229^***^	1.000										
IEPF	0.096^***^	0.139^***^	0.005	1.000									
PPGT	0.607^***^	0.589^***^	0.182^***^	0.110^***^	1.000								
DLFE	0.693^***^	0.414^***^	0.112^***^	0.079^***^	0.871^***^	1.000							
DLFR	0.604^***^	0.375^***^	0.146^***^	0.162^***^	0.597^***^	0.659^***^	1.000						
DLFE _Cent	0.626^***^	0.318^***^	0.003	0.053	0.553^***^	0.614^***^	0.406^***^	1.000					
DLFR _Cent	0.429^***^	0.437^***^	0.101^***^	0.094^***^	0.450^***^	0.408^***^	0.366^***^	0.414^***^	1.000				
ETDZ_Cent	0.348^***^	0.677^***^	0.218^***^	0.060^*^	0.385^***^	0.278^***^	0.343^***^	0.242^***^	0.195^***^	1.000			
Urbpopu	0.441^***^	0.508^***^	0.173^***^	0.137^***^	0.590^***^	0.437^***^	0.344^***^	0.367^***^	0.405^***^	0.499^***^	1.000		
School	0.429^***^	0.437^***^	0.101^***^	0.094^***^	0.450^***^	0.246^***^	0.408^***^	0.366^***^	0.414^***^	0.195^***^	0.405^***^	1.000	
WL	0.693^***^	0.414^***^	0.112^***^	0.079^**^	0.671^***^	0.266^***^	0.659^***^	0.614^***^	0.408^***^	0.278^***^	0.437^***^	0.408^***^	1.000

*p < 0.1; **p < 0.05; ***p < 0.01

## Spatial patterns of IPRs protection

Table 4 shows the average change in IPRs protection from 2011 to 2020 at national provincial and prefectural levels to demonstrate the general pattern of IPRs protection. There was an average increase in IPRs protection of 0.214 per year at national level, indicating that China has been working on strengthening IPRs protection. Economic globalisation and marketisation have resulted in greater industrialisation (He et al., 2014), and industrial development therefore needs to be protected by stronger IPRs institutions. Table 2 also shows that the economic growth figures for Jiangsu, Shandong and Anhui were 8.2%, 9.3% and 5.4%, respectively, while that of Henan, which is located within the central part of China, was only 4%. Henan and Anhui experienced much lower rates of growth, because they have no geographical advantages and do not contain important trading ports. These figures reflect the fact that growth was unequal across the HEZ. Within each province, Yancheng (0.291), Suqian (0.217), Huaian (0.198) and Xuzhou (0.193) were the top four cities in terms of growth, all of which are located in Jiangsu province. In addition, Lianyungang (0.099) and Rizhao (0.101), which are important trading ports, ranked among the cities with the highest growth rates. These results once again indicate that the closer cities are to coastal regions, the faster IPRs protection increases in strength.

**Table 4:** Average change in IPRs protection per year in HEZ

	IPRs protection strength	
	Change	%
China as a whole	0.214	12.331
Jiangsu Province	0.223	8.232
Xuzhou	0.193	6.227
Suqian	0.217	7.315
Lianyungang	0.099	4.362
Huaian	0.198	7.723
Yancheng	0.291	7.309
Shandong Province	0.260	9.322
Jining	0.109	4.874
Heze	0.092	3.945
Linyi	0.086	3.827
Zaozhuang	0.089	3.631
Rizhao	0.101	4.679
Taian	0.082	3.943
Henan Province	0.164	4.011
Shangqiu	0.059	3.181
Kaifeng	0.107	5.151
Zhoukou	0.094	4.002
Anhui Province	0.199	5.976
Huaibei	0.069	2.993
Suzhou	0.134	4.871
Fuyan	0.084	3.487
Bengbu	0.185	5.365
Bozhou	0.106	4.003

**Fig. 3:**
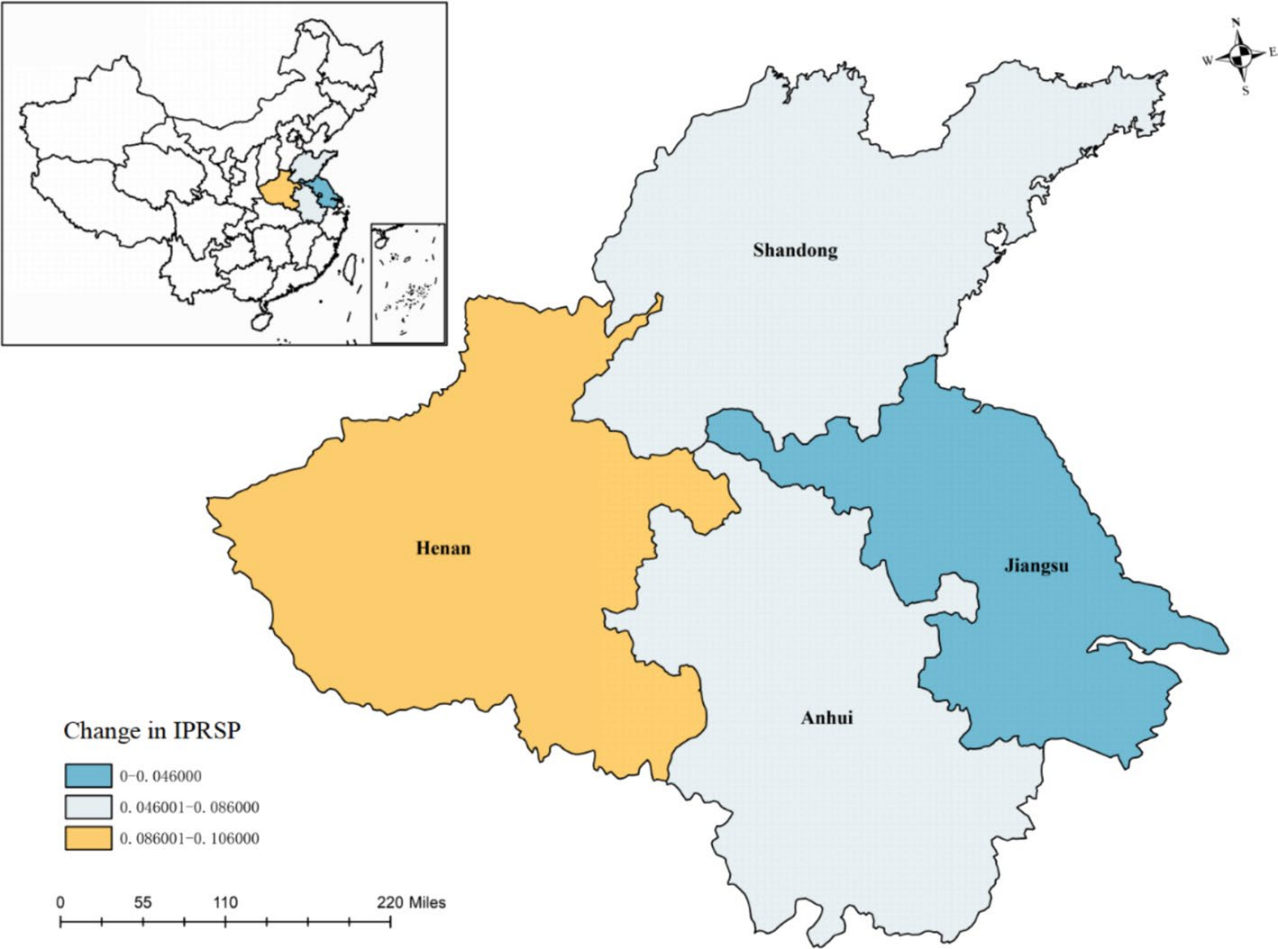
Spatial distribution of IPRs protection at provincial level

Figure 3 illustrates the provincial-level distribution of IPRs protection. When examining the average annual figures, a discernible pattern of variation across the eastern and western regions within the four provinces encompassed by HEZ becomes apparent. Notably, Jiangsu, which is geographically positioned towards the easternmost end of this zone, has the lowest level of IPRs protection. Conversely, Henan, situated towards the westernmost boundary of the zone, exhibits the highest level of IPRs protection. This spatial distribution indicates a significant disparity in the level of intellectual property rights safeguarding measures implemented across the provinces, with a clear east–west gradient distinguishing these variations. Gao and Zhai (2021) found that decentralisation has a similar spatial effect to the spatial pattern observed in our study. This strongly supports the theoretical construction and empirical basis of this paper.

**Fig. 4:**
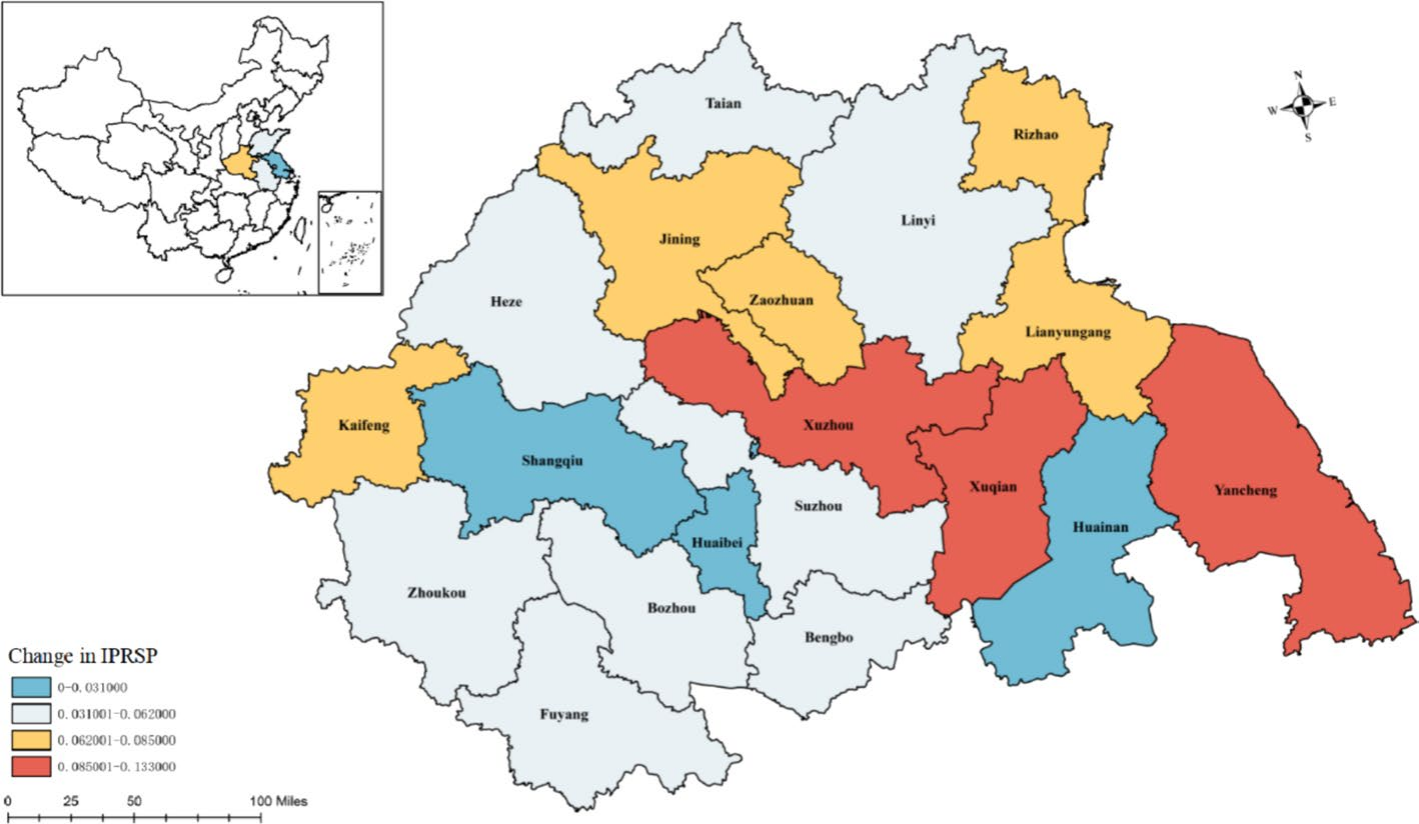
Spatial distribution of IPRs protection at prefectural level

There are substantial spatial disparities between prefectures regarding changes in intellectual property rights (IPRs) protection, as vividly illustrated in Fig. 4. The spatial pattern at the prefectural scale is predominantly characterised by a high level of eastward concentration and a relative dearth in the western regions. This geographical dichotomy is quite pronounced. A total of three cities are positioned in the first tier, which signifies that IPRs protection is of the utmost importance in these cities and they have advanced levels of IPRs protection, while an additional eight cities are classified in the second tier, indicating that they have a respectable level of IPRs safeguarding. In stark contrast, the high-value region, encompassing the third and fourth echelons of IPRs protection, also consists of a total of eight cities. This underscores the complexity of intellectual property rights protection in China and the need for region-specific strategies to ensure equitable and robust IPRs protection across the board.

In addition, we conducted hotspot analysis using local spatial statistics (Moran’s I), and the Getis-Ord ${G}_{i}^{*}$ statistic (Ord, and Getis, 1995), to explore a clustering pattern of IPRs protection that was observed at the prefectural level. The Global Moran’s I provides an overall description of spatial autocorrelation across the study area, while Getis-Ord ${G}_{i}^{*}$ provides a detailed analysis of hotspots and coldspots within a specific region (Ord, and Getis, 1995). Thus, if changes in IPRs protection within a city are substantial and that is also the case for its neighbouring cities, then it is a hotspot.

**Fig. 5:**
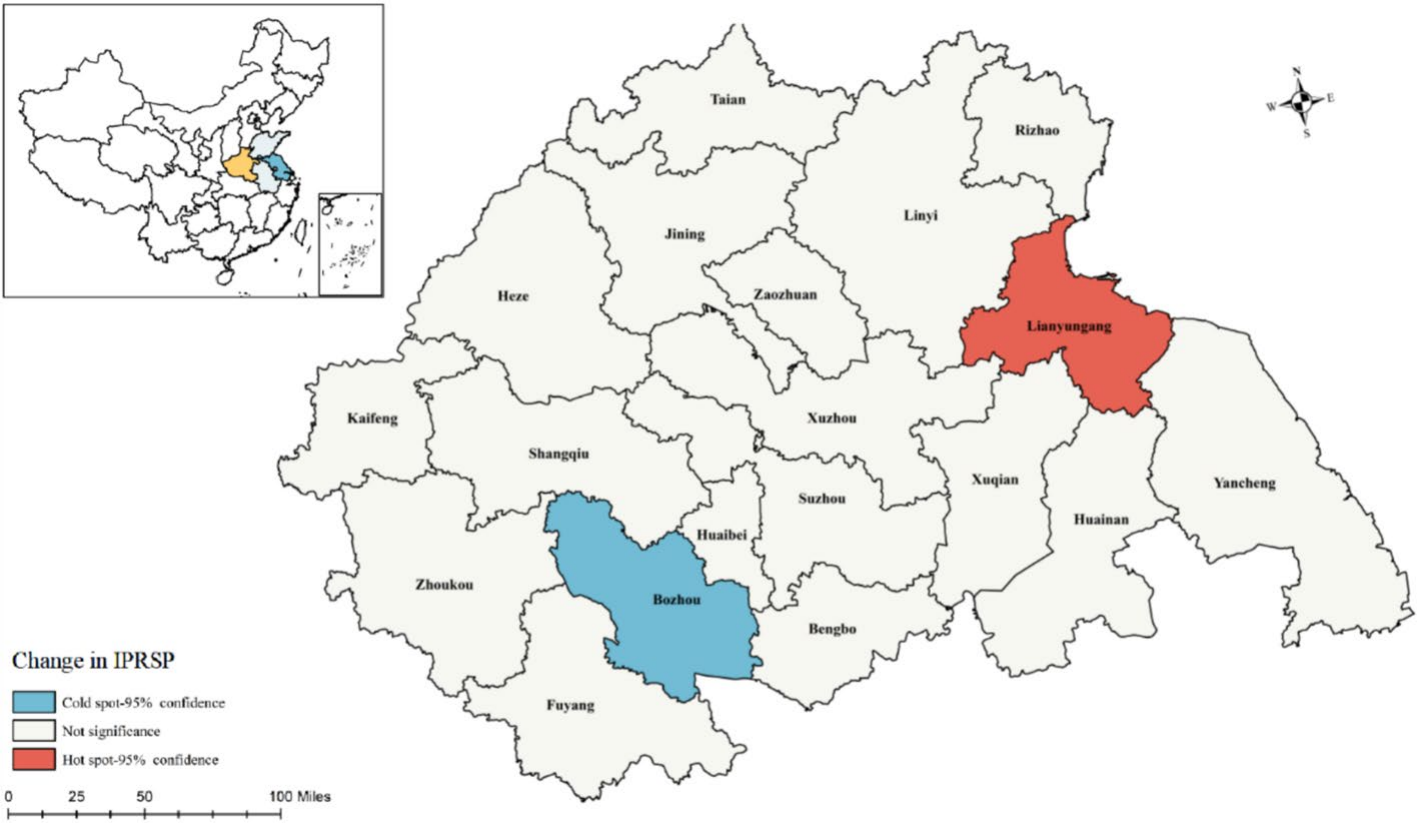
Hotspot analysis of IPRs protection in HEZ

First, Table 5 presents the results of the Global Moran’s I. The positive results obviously indicate the existence of positive spatial autocorrelation, which provides a basis for further spatial analysis. The hotspot analysis shows the spatial inequalities within a specific region (Fig. 5). The IPRs protection hotspots are mainly concentrated in Lianyungang which is in Jiangsu province. Lianyungang is a coastal city with the advantage of harbour traffic, which is conducive to attracting foreign investment. Specific regional regimes affect the location choices of foreign firms (Meyer & Nguyen, 2005). Local governments wanting to profit from Lianyungang's favourable geographical location will make great efforts to create a liberal environment for foreign investment. This affects FDI spillovers by influencing the willingness of foreign firms to transfer, develop and protect technology, the absorptive capacity of local firms, and the way in which the two types of firms interact with each other (Yi et al., 2015). IPRs will be strengthened by this process as it will continue to provide incentives for foreign investment.

**Table 5:** the results of Global Moran’s I in HEZ

Year	(1)Moran’s I	(2)P-value
2011	0.104	0.000
2014	0.041	0.000
2017	0.061	0.000
2020	0.084	0.000

In 2016, Lianyungang promulgated 85 policies on IPRs, second only to Xuzhou, and the city’s average number of policies grew by 61% from 2010 to 2020, which means that it ranks as number one among the 20 cities studied. These policies are mainly concerned with the market order relating to IPRs and patent marketisation. In addition, Lianyungang has promoted the patent pool operating model to develop IPRs intensive industries, whereby two or more patentees agree to license one or more of their patents to the other parties (Li et al., 2016). This suggests that the increase in IPRs protection in Lianyungang is due to the threefold process of economic transition.

## Results and discussion

### OLS estimation

The model used in this study may contain a multicollinearity issue. For example, the FDI and Trade variables are percentage values based on GDP. In addition, the average wage per capita (WL) also has an impact on innovation, and thus WL may also influence patent applications (Weinhold & Nair-Reichert, 2009). Thus, in order to test whether multicollinearity exists, we ran collinearity diagnostics and tests using Pearson's correlation coefficients. The VIFs of all the independent variables were less than 5, and the largest Pearson's correlation coefficient in the model was 0.671. Thus, the statistical results demonstrate that all the independent variables are relatively independent and that our estimations are unaffected by the multicollinearity issue. Overall, IPRs protection in HEZ is not always positively related to decentralisation, marketisation and globalisation.

In Table 6, an R^2^ value of 0.856 indicates that the OLS model at prefectural level was a good fit. Furthermore, the adjusted R^2^ value is relatively large, which also suggests that this does not result from the inclusion of a large number of independent variables. The fitness of the OLS model at the prefectural city level reveals that IPRs protection can be explained by the theoretical framework of economic transition. FDI was used to represent globalisation, and has a negative effect on IPRs protection, indicating that globalisation negatively affected IPRs protection. This conclusion also verifies Grossman and Helpman’s (1993) views. IEPF and PPGT represent marketisation, and both have positive and significant effects on IPRs protection, indicating that the increase in the innovation expenditure of private enterprises and patents granted to private enterprises can serve to enhance IPRs protection. This is because the market value of innovative outputs needs to be guaranteed. DLFE and DLFR were used to represent decentralisation and both had significantly positive impacts on IPRs protection, especially DLFE. One possible explanation for this is that government finance improves IPRs protection by strengthening interregional cooperation (Naim, 2020). Overall, the results of the OLS modelling demonstrate that the threefold process of economic transition can explain the changes in IPRs protection.

**Table 6:** Results of OLS Modelling

Globalisation		Marketisation		Decentralisation		Control variables	
FDI	− 0.03^5***^	IEPF	0.294^**^	DLFE	0.410^***^	Urbpopu	0.035^*^
Trade	0.076	PPGT	0.072^*^	DLFR	0.244^**^	School	0.028
Coasal_City	− 0.042			ETDZ	0.033^*^	WL	− 0.282
				Central	− 0.272	Base	0.176^**^
R^2^	0.856						
Adjusted R^2^	0.781						

*p < 0.1; **p < 0.05; ***p < 0.01

### Multi-level and autocorrelation analysis

Table 7 displays the results of the multi-level analysis. Testing the R^2^ and adjusted R^2^ values enabled us to establish whether multi-level modelling is more effective than other types of modelling. The R^2^ and adjusted R^2^ values for Model 2 were 0.053 and 0.082 higher than those for Model 1, respectively, indicating that multi-level modelling was a better fit. In addition, we used the Bayesian Information Criterion (BIC) to evaluate the goodness-of-fit of the multi-level modelling. The BIC values reflect the quantified degree of improvement in the two-level models as opposed to the one-level mode, and smaller BIC values indicate that the models are more accurate (Cheung et al., 2006). As the BIC value of Model 2 (3598) is smaller than that of Model 1 (3721) by an amount of 123, we concluded that multi-level analysis improves the accuracy of the modelling and that levels of administrative hierarchy and spatial scale do affect IPRs protection. The F-statistic is used to assess the overall significance of a regression model, and thus to determine whether the model, as a whole, is a good fit for the data.

**Table 7:** Estimations of multi-level analysis

	Change in IPRs protection		
	Model 1	Model 2	Model 3: autocorrelation
Prefectural level			
FDI	− 0.331^***^	− 0.283^**^	− 0.254^*^
Trade	0.159^*^	0.565^***^	0.446^***^
Coastal_City	− 0.015	− 0.025	0.034
IEPF	0.235^*^	0.4444^***^	0.515^*^
PPGT	0.091^*^	0.386^***^	0.360^***^
DLFE	0.161^*^	0.119^*^	0.194^***^
DLFR	− 0.147	− 0.021	− 0.036
ETDZ	0.174^**^	0.194^**^	0.231^***^
Central	0.402^*^	0.180^*^	0.294^**^
Provincial level			
DLFE _Cent		− 0.117	0.294
DLFR _Cent		0.149^**^	0.487^**^
ETDZ_Cent		0.124^**^	0.197^**^
SP_Cent		0.165^**^	0.215^**^
Control Variables			
Urbpopu	0.113^*^	0.156^**^	0.228^**^
School	0.079^**^	0.115^*^	0.120^**^
WL	0.042	0.160^*^	0.084^**^
Time effect	YES	YES	YES
Rho spatial lag			0.909^***^
Lambda error			0.871^**^
Moran's I	0.075	0.063	0.051
R^2^	0.696	0.749	0.803
Adjusted R^2^	0.591	0.673	0.755
BIC	3721	3598	3223
F-statistic	7.876^**^	53.442^***^	18.391^**^

*p < 0.1; ** p < 0.05; *** p < 0.01

As a measure of globalisation, FDI still has a negative effect. The results of Model 1 show that the effect of FDI on IPRs is −0.331 and it is significant at the 1% statistical level. This indicates that the inflow of FDI may have a negative impact on IPRs protection at the county administrative unit level. Furthermore, when we added provincial scale variables to construct Model 2, the effect of FDI on IPR diminishes but still remains significant with a coefficient of −0.283, which is statistically significant at the 5% level. Provincial level variables may have had some moderating effect on the relationship between FDI and IPRs, but an increase in FDI is still associated with a decrease in the level of IPRs. In addition, trade has a positive effect on IPRs protection at the 90% and 1% confidence levels in Models 1 and 2 respectively, which may be because, in order to open up the international market, these cities have to abide by the rules of international trade, part of which involves improving IPRs protection. Moreover, the Coastal_City variable does not pass the significance test in Models 1 and 2, indicating that HEZ is not making full use of the geographical advantages of being a coastal city.

Marketisation is measured by the expenditure of private enterprises on patents and innovation. According to Models 1 and 2, IPRs protection has positive correlations with the IEPF and PPGT, particularly the former. Greater demand for innovation from private enterprises and more commercialised patents have the effect of increasing demand for IPRs protection in urban regions. These findings are consistent with the conclusions drawn by Blakeney (2011), who highlighted the positive association between the demand for innovation by individuals and enterprises and IPRs protection. In addition, the market achievements and value of the private enterprises need to be protected by the institution of IPRs, to allow the enterprises to acquire new external debt, generate more sales from new products and produce more innovation patents (Ang et al., 2014).

As a result of the transfer of power, decentralisation has become the key feature of China’s economic transition, and it has significant spatial effects on IPRs protection (Huang et al., 2015). In accordance with Models 1 and 2, four prefectural variables were selected to measure decentralisation, and three of them were found to be significant. DLFE, ETDZ and Central all have positive impacts on IPRs protection. ETDZ is highly significant, indicating that industrial, technological and knowledge agglomeration can strongly promote IPRs protection (Dong et al., 2015). In addition, provincial-level variables were chosen to evaluate the scale effect of decentralisation in the multi-level analysis. These provincial-level variables had a significantly positive effect on IPRs protection, indicating that decentralisation has resulted in a stronger performance. When the provincial-level variables were added, the significance level of these variables in Model 2 increased. For instance, with regard to marketisation, the significance levels of both IEPF and PPGT increased from 90 to 99%, respectively, which again supports the claim that scale effects and the administrative hierarchy play an important role, and that multi-level analysis can offer a better understanding of the IPRs conditions. The control variables all have a positive influence on IPRs protection.

The multi-level regression described in the previous section does not take the effects of autocorrelation into account. As explained previously, it may have an impact on IPRs protection. The significant Moran’s I statistics shown in Table 4 for the residuals in all the models indicates that a potential autocorrelation issue exists. Ignoring this may cause the results to be misleading (Kerkman et al., 2017). According to Table 4, the coefficients of most variables are significant in Model 3 and have the same sign as those for Model 2. Only Coastal_City and DLFE _Cent have the converse coefficients, but neither of them passed the significance tests. In addition, the coefficients of spatial error (lambda) and spatial lag (Rho) are both significantly positive, indicating that the intensity of IPRs protection within a city has a direct effect on other cities, and the spatially correlated omitted variables in Model 3. Meanwhile, the R^2^ and BIC values show that the autocorrelated Model 3 can explain IPRs protection better than the previous multi-level regression.

### Robustness of results

Although the basic aim of the study was to investigate whether the threefold process of economic transition influences IPRs protection, an important caveat to this is the possibility that some forms of endogeneity might exist. This endogeneity could be exacerbated by indices of IPRs protection or economic variables relating to IPRs protection (Allred & Park, 2007) and, in particular, the relationships between IPRs protection and globalisation. Economic development as measured by GDP is one component of the law enforcement-related measure of IPRs protection. In addition, the measure of globalisation is also related to GDP. Thus, its treatment may create an endogeneity issue. On the one hand, it may be expected that regions or cities with strong IPRs protection will increase GDP, which could bias the estimated globalisation coefficients upwards. On the other hand, the estimated globalisation coefficients for regions or cities with a higher level of globalisation than those areas with unfavourable and unobservable characteristics, would be biased in a downwards direction.

Thus, we added instrumental variables into the spatial multi-level analysis. Table 6 shows the two-stage least squares (2SLS) results obtained from applying the instrumental variables to globalisation (only the results of the core variables are shown). Following the method used by Ciccone (2002), the study used the log of land area as the instrumental variable. In addition, based on the work of Roberts and Goh (2011), a further instrumental variable (3-grp) was also selected. The regions or cities were assigned values of 1, 0 or − 1 based on whether their GDP falls in the bottom, middle or top third of the distribution. According to Table 5, unlike the results of the autocorrelation, the estimated elasticity of IPRs protection with respect to globalisation was dramatically increased by adding instruments. In addition, the results also serve to confirm the validity of the instrumental variables. According to Sargan’s test, the hypothesis that the instrumental variables are not related to the disturbance term cannot be rejected. The estimated coefficients of FDI and Trade were − 6.7% and 5.3%, respectively. These imply that FDI is still negatively related to IPRs protection. Similar results were obtained in regard to the provincial level, although the significance and degree of the coefficients clearly increase for the latter (Table 8).

**Table 8:** Estimations of 2SLS based on spatial multi-level analysis

	Prefectural level	Provincial level
FDI	− 0.067^**^	
Trade	0.053^**^	
Coastal_City	0.029	
IEPF	0.714^**^	
PPGT	0.021^**^	
DLFE	5.314^**^	
DLFR	8.055	
ETDZ	0.673^***^	
Central	0.075^**^	
DLFE _Cent		0.179
DLFR _Cent		0.569^***^
ETDZ_Cent		0.263^***^
SP_Cent		0.213^**^
R^2^	0.887	
Adjusted R^2^	0.801	
Instrumental variables	Log(area), 3-grp	
Sargan’s test	1.000	
Significance at first stage	0.000^***^	
Wu–Hausmann test	0.003^**^	
Anselin–Kelejian test	0.987	

*p < 0.1; ** p < 0.05; *** p < 0.01

### Discussion

The negative impact of FDI on IPRs is captured in all the models. This finding echoes a number of studies that have found, from a comparison of developed and developing countries, that IPRs protection is negatively correlated with FDI in countries of the Global South. This may be due to the fact that in these countries, disincentives to imitative behaviour reduce the rate of transfer of new products, thus affecting the level of IPRs protection (Chen, 2021; Helpman, 1993). HEZ represents the low point of China's economic development along the eastern seaboard, and most of the cities in the zones are on the periphery of the economic development pattern seen in the provinces (Tao et al., 2022). Therefore, it is still regarded as relatively backward in economic terms. Moreover, a high quality of FDI will result in greater diffusion and transfer of technology (Assanie & Singleton, 2001), while the differing origins of FDI affect the allocation of aspects of knowledge production and economic growth in the host countries (Fortanier, 2007). The rules of interaction between foreign direct investment (FDI) and intellectual property rights (IPR) vary across Chinese provinces (Yi et al., 2015). This means that regions with differing economic statuses and institutions play different roles in receiving foreign investment. A considerable proportion of FDI in HEZ is concentrated in industries associated with high levels of pollution and high energy consumption. These are not technology-intensive industries, and they do not require advanced technology to manufacture their products. Thus, there is no incentive for FDI to promote IPRs protection in these industries (Lee et al., 2018). However, FDI can promote the development of the local economy and advance the careers of local officials in the short term, because there is fierce competition for capital between regions, and the promotion indicators for Chinese officials emphasise the size of the economy rather than its quality (He et al., 2013). This may explain why FDI has negative effects on IPRs protection in HEZ.

DLFE and DLFR produced completely opposite results at different spatial scales. At the prefecture level, the degree of decentralisation of fiscal expenditure is positively associated with IPRs protection, while the relationship between the degree of decentralisation of fiscal revenue and IPRs protection is not significant. DLFE means that local governments have more autonomy in deciding how to use financial resources (Li & Du, 2021), thus allowing them to allocate resources more flexibly and efficiently. However, local governments with decentralised fiscal revenues are more inclined to invest resources in areas that can bring short-term economic benefits rather than long-term intellectual property rights protection (Zhou et al., 2011). In contrast, DLFE and DLFR produced quite different results at the provincial level. Li and Zhou (2005) found that the central government promotes officials according to a relative economic growth. In order to obtain more political benefits and promotion opportunities, provincial officials will actively compete for more economic resources. Through this process, provincial governments have further incentives to invest in productive projects and reduce IPRs investment. Moreover, under the system of"upward responsibility", some higher-level government bodies have invested in science and technology as a"one-vote veto"indicator for the promotion of lower-level government officials (Bian & Bai, 2017). Local governments will also actively respond to the central government's call to increase IPRs protection policy.

ETDZ shows a robust positive correlation to IPRs, which makes it more important to establish SEZs for IPRs protection. This is similar to the findings of previous studies (Wang et al., 2023). With regard to the control variables, a highly urbanised population means much cheaper transport costs, which has an independent effect on innovation (Weinhold & Nair-Reichert, 2009). Thus, to protect innovative achievements, cities need to promote IPRs protection. Education not only helps to provide innovative human capital, but also enhances awareness of IPRs protection. In addition, we added dummy time variables to the multi-level analysis. The coefficients of these dummy variables showed that IPRs protection has increased over time. Although IPRs protection is not China’s strength, this trajectory is still in line with the policy requirements of different levels of government in China (Frank & Zhang, 2003).

## Conclusions

There is almost universal agreement that IPRs protection is an important facet of innovation and economic growth. The institution of IPRs protection is also viewed as a determinant of the variations in economic growth at different spatial scales. This has led to the creation of a new agenda designed to promote effective interactions between local economic conditions and institutions within the framework of EEG. The study empirically investigated the effects of economic transition on IPRs protection, using HEZ as a sample case, with a particular focus on the spatial diversification of IPRs institutions and economic transition at a local level. Overall, this study found that IPRs protection is not only a national or supranational institution, but also involves disparities at horizontal (between cities) and vertical (between administrative levels) spatial scales, which interact with issues relating to the process of economic transition impacted by EEG.

### Key findings

Overall, the results showed that the threefold process associated with economic transition is significant and demonstrates multiple IPRs protection mechanisms operating at different spatial scales.

First, according to the OLS estimation, in terms of economic transition, marketisation and decentralisation were found to positively influence IPRs protection. This supports the findings of Stinchfield et al. (2013) and Heimeriks and Boschma (2014), who discussed the role of local economic conditions in shaping institutional effectiveness. The results regarding globalisation provide evidence to refute the attempts of the Global North to implement strong IPRs protection on a global scale due to its negative effects. This contrasts with the claims made in the existing literature, such as by Rodríguez-Pose and Zhang (2020), who discussed the positive impact of FDI on IPRs protection.

Second, the spatial multi-level analysis regarding vertical diversification indicates that, unlike at the provincial level, decentralisation has a major influence on IPRs protection at the prefectural level. This finding is in line with the rationale for using the EEG perspective, as discussed by Rodríguez-Pose (2021). More refined conclusions could be drawn by examining the interaction between economic events and political institutions from the perspective of spatial geography. Additionally, according to the dummy time variables, HEZ has become more committed to enhancing IPRs protection over time. These results remained consistent when endogeneity was accounted for by adding instrumental variables. Overall, these findings suggest that incorporating the autocorrelation impacts of the multilevel regression influences the estimation outcomes, as supported by Crouch and Voelzkow (2009), which in turn may have a profound effect on regional and urban IPRs protection and economic planning.

Third, under the guidance of the EEG framework, we used GIS technology to show the horizontal diversification of IPRs protection on a multi-level spatial scale. The spatial analysis revealed horizontal unevenness in IPRs protection between regions and cities, which aligns with Boschma et al.’s (2017) findings, and vertical differences between different administrative levels, as noted by Beer et al. (2019). The hotspot analysis highlighted a detailed pattern of institutional spatial inequalities, with IPRs protection hotspots being mainly concentrated in Lianyungang.

### Implications

#### Theoretical implications

The theoretical significance of this study is as follows: Firstly, economic transformation and intellectual property rights protection were explored in relation to two dimensions under the EEG framework, thus showing how the new field of urban geography affect intellectual property rights protection. Horizontally, we compared cities located within the same HEZ, while vertically, we distinguished between regions at different administrative scales. These two dimensions have been shown to be important (Gokan et al., 2019). With regard to economic transition, we believe that the geographical location of a city or region in different dimensions is also an important determinant of local IPRs institution (He et al., 2008). This aligns with the findings of Boschma et al. (2017), who emphasised the significance of regional diversification in the context of economic transition. Moreover, even in a centralised country, such as China, we also support the implementation of an appropriate IPRs institution based on local geographical endowment.

Second, the results obtained for FDI led us to challenge the current assumption that advanced economic development is always positively related to strong IPRs protection, by considering urban geography. Therefore, we believe that the horizontal and vertical spatial diversification of economic transition and IPRs protection shows that the interaction between economic issues and political institutions has specific spatial characteristics at different scales, resonating with the observations of Stinchfield et al. (2013) and Heimeriks and Boschma (2014). More specifically, differences between cities and regions can be regarded as horizontal spatial differences, and vertical spatial differences are the result of dispersion.

Third, this study helps to narrow the gap in previous literature on the relationships between economic transition and IPRs protection by identifying the value of hierarchy and space in institutional research. This is consistent with the arguments of Beer et al. (2019) and Rodríguez-Pose (2021), who emphasised the need to consider spatial and hierarchical factors in understanding institutional effectiveness. These can be used to overcome the administrative barriers associated with conflicts and disparities between local interests and achieve more effective cooperation and coordinated development between regions. Therefore, our study helps to determine which factors affect IPRs protection in different spaces and at different levels.

#### Managerial implications

Managerially, this study highlights the value of the EEG framework in enhancing understanding of how economic transition affects institutions. We argue that the analytical approach and empirical strategy of spatial geography employed in this study provide effective tools for assessing technology and innovation-related issues. The study advocates a greater focus on the role played by IPRs protection at the sub-national level in regard to regional planning and development, especially in less-developed regions and particularly the Global South. Thus, we propose the following policy recommendations.

Economic and technological development zones and industrial parks can affect the creation of regional IPRs protection. Thus, it makes sense to allocate a more generous proportion of land to industrial parks. In addition, the public finance and tax system needs to be redesigned to increase the government's income from innovations, so as to reduce the dependence of local governments on land rental income.

The findings with regard to decentralisation indicate that meeting the needs of political interests is an important task for China’s urban governance, using public funding, distributed via the government's fiscal and tax system. However, using the government's fiscal and tax system to improve innovation capability is not a focus of local government bodies. Instead, political interests are the fundamental driving force behind innovation in Chinese cities.

### Limitations and directions for future research

Our study has some shortcomings with respect to the methodological design. Although the study considered the geographical characteristics of the IPRs institution, it did not captured its path dependence in space. Furthermore, our study only regards the IPRs institution as a static measurement, and does not take into account the fact that it will dynamically flow between regions with the mobility of production factors. In addition, while ETDZ captures clustering and/or localised economies to some extent, it is a somewhat crude measure compared to urban population.

Thus, future research could use path analysis to consider the path dependence of the IPRs institution in space and within the administrative hierarchy. It could also explore on the dynamic mobility of the IPRs institution by analysing its space–time mechanism simultaneously. Further studies could also be conducted that involve integrating a measure of localisation, that can be connected to knowledge spillovers and knowledge leakage risks and thus IPRs institutions.

## Data Availability

The data that support the findings of this study are available on request from the corresponding author. The data are not publicly available due to privacy or ethical restrictions.
